# Advancing physiology through transparency: Celebrating our first registered report

**DOI:** 10.1113/EP091963

**Published:** 2024-10-14

**Authors:** Peter Rasmussen, Michael J. Tipton, Alex Stewart, Damian M. Bailey

**Affiliations:** ^1^ Genmab A/S Valby Denmark; ^2^ Extreme Environments Laboratory, School of Sport, Health and Exercise Science, Faculty of Science and Health University of Portsmouth Portsmouth UK; ^3^ The Physiological Society London UK; ^4^ Neurovascular Research Laboratory University of South Wales Glamorgan UK

Numerous scientific disciplines face challenges regarding the robustness and replicability of reported results (Wallach et al., 2018). To counter such challenges, new strategies are emerging that aim to improve the rigour and credibility of published research. Preregistration and registered reporting are two of these strategies, with the Open Science movement strongly advocating preregistration of hypotheses and analyses to improve reproducibility and transparency (Nosek et al., 2019). Several platforms allow researchers to share preregistrations [e.g., the Center for Open Science (https://osf.io/), AsPredicted (https://aspredicted.org/), PROSPERO (www.crd.york.ac.uk/prospero) and ClinicalTrials.gov], and some journals, including *Experimental Physiology*, offer registered reports as a format for formal review and publication (https://www.cos.io/initiatives/registered‐reports).

Preregistration involves detailing and publishing a research plan, including hypotheses, design and analysis, before data collection begins. This improves scientific research by separating hypothesis‐testing and hypothesis‐generating analyses, increasing the trustworthiness of results and reducing hidden ‘flexibility’ in data analyses. It helps to avoid improper research practices, such as choosing outcomes based on results or making hypotheses after seeing results. It also combats publication bias by making all planned studies visible, no matter what their results.

Concerns about the cumbersome nature of the registration process for exploratory studies are valid (Richter et al., 2024). Richter et al. (2024) argue that exploratory trials focusing on human physiology should not be categorized as clinical trials, because their objectives differ significantly. Clinical trials are designed to inform regulatory decisions, whereas physiological research aims to uncover underlying mechanisms of the human body. They contend that the International Committee of Medical Journal Editors (ICMJE) guidelines are unsuited for experimental physiological studies, because these studies focus on the adaptability of the human body rather than disease outcomes. Across‐the‐board application of these rigid requirements could discourage innovative and exploratory approaches, stifling scientific creativity and, in turn, discovery and serendipitous findings.

Thus, it is crucial to recognize that all physiology studies, whether exploratory or confirmatory, benefit from pre‐trial registration (Berg et al., 2024). In our view, the ‘registered report’ format adopted by *Experimental Physiology* offers a promising solution by allowing journals to approve study methods and analyses before data collection. This safeguards methodological quality and transparency without undue restrictions on exploratory efforts. Thus, although regulatory requirements should differ between clinical trials and physiological research, pre‐trial registration is an essential practice that should be implemented across all types of studies. To that end, registered reports not only increase publication rates with very limited additional work, because the protocol is already written, but also ensure methodological quality assessment and transparency.

Moreover, registered reports handle both hypothesis‐testing and hypothesis‐generating research, addressing concerns about mistaking exploratory physiology studies for clinical trials. Regardless of the type, by securing principal acceptance based on the methodology rather than outcomes, registered reports ensure that the study design, hypotheses and analysis plans are thoroughly assessed by peer reviewers for methodological quality before any data are obtained (rather than simply registered, regardless of quality). The main benefit of registered reports is that the results are published no matter the outcome of the study, providing the researchers follow their approved methods or transparently report any deviations. This approach highlights the importance of methodological excellence over the novelty or direction of findings. The detailed peer review aspect of a registered report has the added advantage of giving high‐quality methodological input at an early stage of the research, when it can have the most significant impact. This could be a particularly important resource for early‐career researchers. Furthermore, a published protocol can be cited in later publications, raising the profile and influence of the work among scientists. This visibility boosts the trustworthiness of the research and helps the dialogue and improvement of best practices in the field (Field et al., 2020).

Preregistration requires researchers to decide in advance how they will design, collect and analyse data, although organizations such as the Open Science Framework (OSF) and Declare Design have helpful tools and workflows to aid this. Honesty is important. Reporting changes from the preregistered plan, explaining why, and sharing materials and data are necessary for retaining credibility. This honesty also promotes intellectual humility, enabling researchers to see mistakes as opportunities to improve rather than negative threats. Submitting a research protocol for peer review encourages researchers to incorporate interdisciplinary perspectives before a study is conducted, taking on different and complementary views to help solve a complex question. This process often involves collaboration with experts such as statisticians and epidemiologists, whose skills are crucial for designing robust studies and analysing complex data. Although there might be initial hesitation to involve, for example, statisticians, owing to concerns about their depth of knowledge in specific areas, these interdisciplinary collaborations enhance methodological rigour and ensure the statistical integrity of research. By engaging with a broader range of expertise, researchers can address potential issues more comprehensively, leading to more reliable, widely applicable and valid outcomes, ultimately strengthening the overall quality of the study.

At *Experimental Physiology*, we support registered reports and want to improve how research is constructed, assessed, reported and incentivized. This editorial affords a timely opportunity to celebrate the full (i.e., Protocols and Results) publication of our first registered report (Farrow et al., 2024). To the best of our knowledge, this is the first registered report in the field of physiology to have both its Protocol (Farrow et al., 2021) and Results published in the same journal.

We have offered the registered report publishing format since 2020 and remain one of the few physiology journals to do so (https://www.cos.io/initiatives/registered‐reports#journals). However, since its introduction, the take‐up among authors has been lower than we hoped. This might be the result of some common misconceptions about the format, which we address below.

- Limited to hypothesis‐driven research: Registered reports are suitable for both hypothesis‐testing and exploratory studies, because exploration also benefits from careful planning, proper design, methodology and analysis.
- Too rigid and inflexible: Registered reports are sometimes thought to be rigid, but deviations from the preregistered plan are permitted if transparently reported and justified.
- Only for certain disciplines: Registered reports are not limited to fields such as psychology or pharmaceutical research; they are applicable across a wide range of disciplines.
- Time‐consuming and bureaucratic: The registered report process is seen by some as time‐consuming and bureaucratic. Although it requires thorough planning and peer review, it ultimately enhances research quality and credibility. In the long term, it is likely to save resources. Early‐stage peer review should prevent conducting, with associated costs, methodologically unsound research. Furthermore, the publication of these studies, regardless of their findings, might prevent other groups from unwittingly ‘re‐conducting’ studies that might have negative findings and will also ensure that resource is not invested by groups looking to replicate or expand upon ‘interesting’ but irreproducible findings.
- Inhibits scientific creativity: The structured nature of registered reports is feared to stifle creativity. However, registered reports can enhance creativity by encouraging interdisciplinary thinking in study design and analysis plans.
- Difficult to implement: Registered reports are not difficult to implement and do not require specialized knowledge. Many resources and guidelines are available to help researchers navigate the registered report process.
- Ideas being stolen: There is concern that publishing a Protocol might lead to theft of ideas or intellectual property. However, we contend that this initial publication, which is not implemented by all journals that have adopted the registered report format, provides a time‐stamped record of an original idea, protecting against intellectual theft and establishing primacy of research methods and hypotheses.
- Publishing the protocol is redundant: Publishing the Protocol not only time stamps the authors’ research concept but is an important means of communicating the availability of this innovative format to our wider research community. It also brings benefits from an accountability perspective, publicly holding the journal in question to the promise of in‐principle acceptance of the subsequent Results article.

In summary, preregistration is a great way to make scientific research more rigorous and reliable and to improve its quality. As more researchers use this practice, the scientific community will get closer to its main goal: creating strong, reproducible knowledge. Registered reports, as offered by *Experimental Physiology*, provide an opportunity for authors and the field of science as a whole. The assurance of publication of methodologically sound research before results are known reduces publication bias and enhances the visibility of all rigorously conducted studies, including those with negative or null results. For researchers, this means greater confidence in investing time and resources into studies that, regardless of the outcome, will contribute to advancing the field of physiology. A schematic summary of the concepts, programmatics and benefits underpinning Registered reports is illustrated in Figure 1, and more detailed formal submission details can be found here (https://ep.msubmit.net/cgi‐bin/main.plex?form_type=display_requirements#registered). We look forward to receiving more submissions to this category.

**FIGURE 1:**
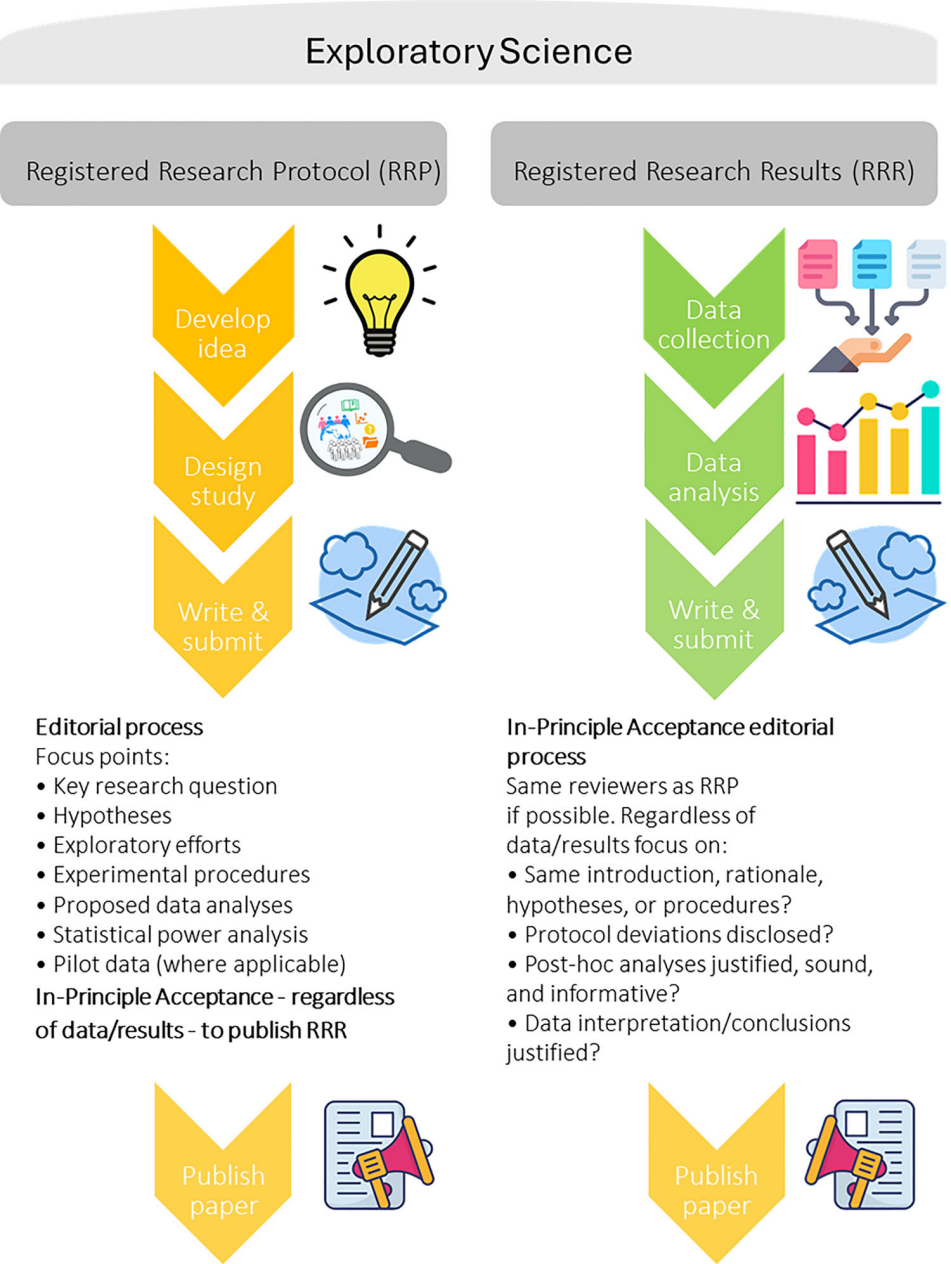
Registered reports workflow. The concepts, programmatics and benefits, from idea to publication. The process allows for flexibility for ad hoc or exploratory analysis, as long as this is documented in the final report.

## AUTHOR CONTRIBUTIONS

Conception or design of the work: Peter Rasmussen, Damian M. Bailey, Alex Stewart and Michael J. Tipton. Drafting of the work or revising it critically for important intellectual content: Peter Rasmussen, Damian M. Bailey, Alex Stewart and Michael J. Tipton. All authors approved the final version of the manuscript and agree to be accountable for all aspects of the work in ensuring that questions related to the accuracy or integrity of any part of the work are appropriately investigated and resolved. All persons designated as authors qualify for authorship, and all those who qualify for authorship are listed.

## CONFLICT OF INTEREST

DMB is Editor‐in‐Chief of Experimental Physiology, Chair of the Life Sciences Working Group, member of the Human Spaceflight and Exploration Science Advisory Committee to the European Space Agency and member of the Space Exploration Advisory Committee to the UK and Swedish National Space Agencies. DMB is also affiliated to Bexorg, Inc. (USA) focused on the technological development of novel biomarkers of cerebral bioenergetic function and structural damage in humans PR is Senior Editor of Experimental Physiology.

## FUNDING INFORMATION

DMB is supported by a Royal Society Wolfson Research Fellowship (#WM170007).
